# ViT-Based Face Diagnosis Images Analysis for Schizophrenia Detection

**DOI:** 10.3390/brainsci15010030

**Published:** 2024-12-29

**Authors:** Huilin Liu, Runmin Cao, Songze Li, Yifan Wang, Xiaohan Zhang, Hua Xu, Xirong Sun, Lijuan Wang, Peng Qian, Zhumei Sun, Kai Gao, Fufeng Li

**Affiliations:** 1 School of Traditional Chinese Medicine, Shanghai University of Traditional Chinese Medicine, Shanghai 201203, China; huilin_liu24@shutcm.edu.cn (H.L.); wanglj@shutcm.edu.cn (L.W.); qianpeng@shutcm.edu.cn (P.Q.); 1506@shutcm.edu.cn (Z.S.); 2 State Key Laboratory of Intelligent Technology and Systems, Department of Computer Science and Technology, Tsinghua University, Beijing 100084, China; crm23@mails.tsinghua.edu.cn (R.C.); 2023114032@stu.hebust.edu.cn (S.L.); 2023114119@stu.hebust.edu.cn (Y.W.); 2023114105@stu.hebust.edu.cn (X.Z.); 3 School of Information Science and Engineering, Hebei University of Science and Technology, Shijiazhuang 050018, China; gaokai@hebust.edu.cn; 4 Shanghai Pudong New Area Mental Health Center, Tongji University, Shanghai 200124, China

**Keywords:** schizophrenia detection, face diagnosis images, Vision Transformer (ViT), clinical facial features analysis

## Abstract

Objectives: Computer-aided schizophrenia (SZ) detection methods mainly depend on electroencephalogram and brain magnetic resonance images, which both capture physical signals from patients’ brains. These inspection techniques take too much time and affect patients’ compliance and cooperation, while difficult for clinicians to comprehend the principle of detection decisions. This study proposes a novel method using face diagnosis images based on traditional Chinese medicine principles, providing a non-invasive, efficient, and interpretable alternative for SZ detection. Methods: An innovative face diagnosis image analysis method for SZ detection, which learns feature representations based on Vision Transformer (ViT) directly from face diagnosis images. It provides a face features distribution visualization and quantitative importance of each facial region and is proposed to supplement interpretation and to increase efficiency in SZ detection while keeping a high detection accuracy. Results: A benchmarking platform comprising 921 face diagnostic images, 6 benchmark methods, and 4 evaluation metrics was established. The experimental results demonstrate that our method significantly improves SZ detection performance with a 3–10% increase in accuracy scores. Additionally, it is found that facial regions rank in descending order according to importance in SZ detection as eyes, mouth, forehead, cheeks, and nose, which is exactly consistent with the clinical traditional Chinese medicine experience. Conclusions: Our method fully leverages semantic feature representations of first-introduced face diagnosis images in SZ, offering strong interpretability and visualization capabilities. It not only opens a new path for SZ detection but also brings new tools and concepts to the research and application in the field of mental illness.

## 1. Introduction

Schizophrenia (SZ), a complex mental illness, requires accurate detection for effective treatment. With the technological development of artificial intelligence, a large class of techniques that can process large datasets and accurately analyze process large datasets is called computer-aided diagnostics (CADs) [1]. There are two main types of methods for SZ detection based on CAD techniques used: electroencephalogram (EEG) [2,3,4] and brain Magnetic Resonance Imaging (MRI) [5,6,7]. EEG rhythms intricately reflect a range of physiological and cognitive processes. Each band of EEG signals represents a different spectrum of electrical activity in the brain, and these classifications are fundamental in EEG analysis, providing insights into cognitive states and neurological conditions. Luján et al. [8] combined machine learning algorithms and EEG signals obtained using a 32-channel helmet to analyze high temporal resolution information from the brain for SZ detection. Agarwal et al. [9] designed an SZ detection system using a fast Fourier transform to capture and analyze local variations in the EEG signal. Additionally, Mudholkar [6] employed and analyzed 46 patients’ subcortical regions and ventricular areas of brain MRIs for contributing most to the prediction of SZ.

Previous works for SZ detection that required a lot of data used biomedical signals of an EEG and all the slices of the brain MRI images reflecting all viewing perspectives. However, acquiring EEG or MRI data from potential SZ patients is a tedious process that probably causes the patient to be uncooperative and even reluctant to be detected, making it challenging to obtain the necessary information. This tedious process can lead to patient reluctance and uncooperative behavior, further complicating the diagnostic process. These challenges can hinder the practical application of such methods in clinical settings.

Hence, finding a new method that reduces invasiveness and time costs while enhancing diagnostic accuracy is particularly crucial. Face diagnosis in traditional Chinese medicine (TCM) is a unique diagnostic method that judges the health condition of the body by observing the characteristics such as spirit, complexion, shape, and state of the face without interfering too much with patients. Compared to EEG and MRI, face diagnosis is non-invasive, cost-effective, and easy to apply, making it a promising alternative for widespread clinical use. Yang et al. [10] collected TCM face diagnosis images of depression patients and healthy controls, extracted and analyzed the differences in the objective parameters of the face, and found that depression patients with liver stagnation and spleen deficiency syndrome have different face characteristics. Meanwhile, the spirit and expression of the face are the external manifestations of mental activities such as spirit, consciousness, and thinking [11].

Therefore, face diagnosis images can be an important window to understand mental and psychological conditions. They are uniquely suited to the diagnosis of mental illness, capturing subtle emotional expressions and nonverbal characteristics of patients. Observation of facial information and recognition of their changes are critical to the accurate diagnosis of mental illness [12,13]. To highlight the advancements of the proposed face diagnosis image-based method over traditional SZ detection techniques, Table 1 compares EEG, MRI, and face diagnosis images in terms of invasiveness, clinical interpretability, patient compliance, and key advancements.

In recent years, deep learning and computer vision techniques have shown promising results in face diagnosis image analysis, as they can automatically learn relevant feature representations for various issues. Some researchers [14,15,16] have reported the effectiveness of deep learning models in detecting some mental illnesses through the analysis of these subtle emotional expressions. For instance, Chandra et al. [17] compared EfficientNet-B2 and VGG19 to assess a person’s mental condition and validated that facial emotion recognition plays a huge role in mental illness detection. Li et al. [18] proposed a two-stream model SFTNet for identifying depression based on micro-expressions, which learns feature representations from face diagnosis images at a single time node.

Among deep learning techniques, Vision Transformer (ViT) has gained prominence for its powerful ability to learn comprehensive feature representations from visual data [19]. ViT models utilize the self-attention mechanism to effectively learn deep semantic feature representations and long-term dependencies from images, excelling in various image recognition tasks. Lan et al. [20] adapted a ViT model to analyze chest X-ray images for capturing long-term dependencies, exploring correlations, and learning features with richer semantic information. Marcos et al. [21] used a pure ViT to learn the contextual relationships between low-dose computed tomography image patches and preserve spatial details of medical images. Dixon et al. [22] and Qiu et al. [23] utilized a ViT model to capture long-range, global dependencies between features, which is beneficial for analyzing the medical image feature. By applying ViTs to face diagnosis images, we can learn semantic feature representation relevant to SZ detection, offering high detection accuracy.

Meanwhile, in order to better explain the relationship between face diagnosis images and SZ disease, a visual method is applied to analyze the clinical facial information. Admittedly, many researchers [24,25,26] have utilized Grad-CAM [27] to focus on the gradient between the output category scores and the input image to identify important regions, but it is typically effective when applied to convolutional neural networks (CNNs) where spatial features are directly processed. On the other hand, attention rollout [28] aligns better with the internal workings of ViTs, which are chosen in our paper and belong to self-attention mechanisms without explicit spatial hierarchies, so it is used to visualize the face diagnosis images. This method can aggregate the attention weights across multiple heads and layers of the ViT, effectively tracing the flow of information throughout the network.

The method proposed in this paper is the first to apply ViT models in learning semantic feature representations from face diagnosis images for SZ detection and employ attention rollout in visualizing these features of SZ. The method acquires and analyses these semantic feature representations in SZ detection with more accurate detection and more interpretable results. Our contributions are as below as follows:

1. Face diagnosis images are introduced into the deep learning field of SZ detection.
2. A pre-trained ViT model is utilized to learn semantic feature representation of face diagnosis images with SZ and to predict the SZ detection results.
3. Attention rollout is applied to visualize facial semantic feature representations relevant to SZ and to analyze the importance of semantic feature representations representing detection results to different clinical facial features.

The rest of this paper is organized as follows: In Section 2, current related work is introduced. In Section 3, the proposed method is discussed in detail. Section 4 evaluates the proposed method and discusses the experiment results. Finally, the important conclusion is given in Section 5.

## 2. Related Work

Facial semantic feature representations have been used for the detection of various psychiatric disorders, but their effectiveness in the detection of SZ remains to be explored. ViTs have excelled in image analysis to capture global features. Image visualization technology has helped researchers and clinicians intuitively understand model decisions and verify models’ effectiveness. These studies can contribute to the development of SZ detection.

### 2.1. Face Diagnosis Image Analysis for Psychiatric Disorders

Face diagnosis image analysis holds great potential in detecting psychiatric disorders. Early research primarily focused on using facial expressions in images to predict emotional states, thereby inferring the presence of psychiatric disorders. Aina et al. [29] propose a hybrid architecture for mental disorder detection by analyzing facial expressions. Kumar et al. [30] utilized facial expression-based emotion recognition analysis for the detection of depression symptoms along with their severity level assessment. In contrast, our approach directly learns semantic feature representations from face diagnosis images rather than relying on emotion recognition for psychiatric disorder detection.

### 2.2. Vision Transformers in Medical Image Analysis

ViTs emerged as a powerful alternative to the traditional CNNs for image analysis tasks. Originally proposed by Dosovitskiy et al. [19], ViTs have shown superior performance in various image classification benchmarks. Unlike CNNs, which rely on local receptive fields, a ViT employs self-attention mechanisms to capture global context across the entire image, leading to better feature representation learning. The advantage aligns closely with the goals of this study, where learning complex, global facial feature representation is essential for SZ detection.

Recent advancements in ViTs have demonstrated their effectiveness in analyses and diagnoses of medical images. For instance, Tyagi et al. [31] have proposed a ViT model for the classification of pneumonia so that it can reduce the time-consuming chest X-ray evaluation process in far-off places. Huang et al. [20] adapted ViTs for chest X-ray image analysis because they excel at capturing long-term dependencies, exploring correlations, and learning feature representations with richer semantic information. Huang et al. [32] propose an end-to-end ViT-AMCNet with adaptive model fusion and multiobjective optimization for distinguishing the tumor grading of laryngeal cancer pathological images. A ViT module increases the differences between feature representations and improves the de-redundancy. In this work, the ViT architecture is applied in learning semantic feature representation from face diagnosis images for SZ detection, aiming to exploit its strength in capturing detailed visual patterns relevant to psychiatric conditions.

### 2.3. Visualization for Face Diagnosis Image Analysis

Visualization techniques play a crucial role in interpreting the results of disease detection. By highlighting the regions of an image that contribute most to prediction results, visualization techniques help in understanding the detection process of models. Lin et al. [33] observed and analyzed the importance of seven different facial regions in detecting coronary artery disease. Hong et al. [24] generated a facial heatmap for visual comparison of model results. This paper combines the ViT model with attention rollout to visualize the key facial regions that are indications of SZ. This not only enhances the interpretability of our model but also provides valuable insights for clinical diagnosis.

## 3. Materials and Methods

**Figure 1:**
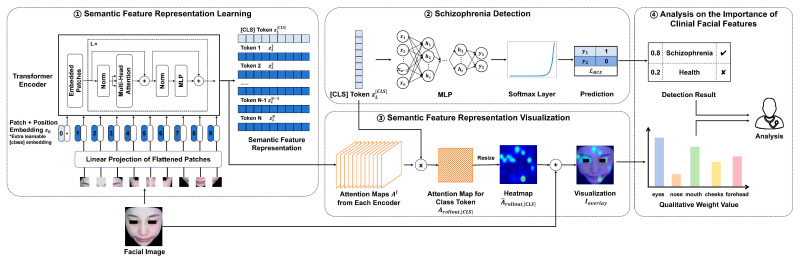
An overview of the model architecture of our method. The framework consists of four main components: (1) learns facial semantic feature representations based on ViT structure; (2) inputs the class token into MLP with BCE loss function to obtain detection results; (3) employs and calculates the attention maps from transformer encoders to visualize facial semantic feature representations; (4) quantifies the importance of clinical facial features based on detection and visualization results.

In this section, the proposed method will be introduced in detail, including how facial semantic feature representations are learned and are then applied in detection and how key semantic features of face diagnosis images are visually interpreted. After these, the importance of different facial regions in SZ detection can be quantified by calculating their weight value with a self-attention mechanism. Figure 1 presents the framework proposed.

### 3.1. Semantic Feature Representation Learning

A pre-trained ViT model is employed for semantic feature representation learning of face diagnosis images. The semantic feature representation learning model is mainly composed of two modules, including the patch embedding layer and the transformer encoder module. These two core modules will be introduced in detail below.

The patch embedding layer is crucial for converting the input image into a sequence of patches that can be similarly processed to tokens in natural language processing (NLP) tasks. It involves three main steps: image segmentation, image block embedding, and position encoding [34]. First, the input image $X\in R^{H\times W\times C}$ is divided into a specified number of fixed-size patches, where $\left(H,W\right)$ is the input image resolution and *C* is the number of channels. Each patch of resolution $\left(P,P\right)$ is flattened into a one-dimensional vector, resulting in a sequence $X_{p}\in R^{N_{p}\times \left(P^{2}\cdot C\right)}$ where $N_{p}$ is the number of patches and $\left(P^{2}\cdot C\right)$ is the dimension of each flattened patch. Then, through a trainable linear projection layer, these flattened patches are mapped to a *D*-dimensional space, producing patch embeddings $X_{i}^{P}\in R^{D}$, which are more relevant to the current task.

Finally, by merging with fixed and learnable positional embeddings, embeddings of each patch gain positional information to help the model distinguish between different positions. It is worth noting that, for detection, class tokens [CLS] are prepended to the sequence of each patch embedding, and also attached with positional embeddings. After these steps, each embedded patch *z* can be transmitted to the next transformer encoder module as an input.

(1) $$\begin{array}{c} z_{0}=\left[x_{class};x_{p}^{1}E;x_{p}^{2}E;\cdots ;x_{p}^{N_{p}}E\right]+E_{pos}\\ E\in R^{\left(P^{2}\cdot C\right)\times D},E_{pos}\in R^{\left(N_{p}+1\right)\times D} \end{array}$$

The transformer encoder is built by stacking multiple transformer encoder blocks in sequence. Each block refines the embeddings from the last patch embedding layer. Simply put, transformer encoder blocks are composed of Layer Normalization (LN), a multi-head self-attention mechanisms module (MHSA), and a multi-layer perceptron module (MLP). A residual connection is applied after each block.

**Figure 2:**
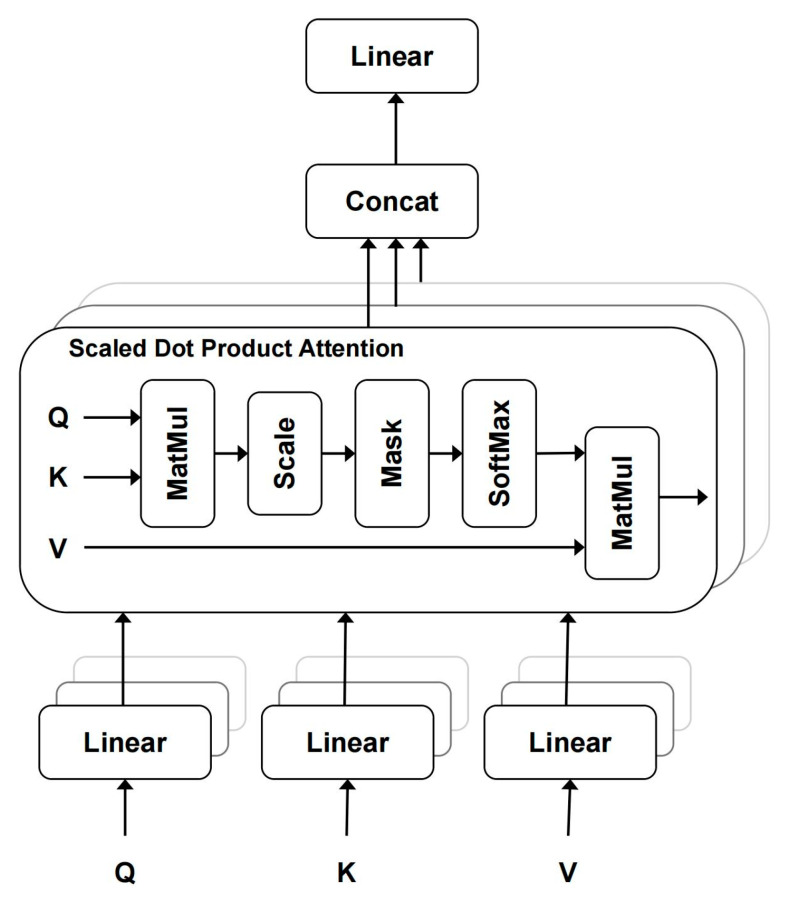
Self-attention module. k self-attention operations are run in parallel and project their concatenated outputs. The self-attention operation is based on the query vector *Q*, and the weight distribution is obtained by calculating the similarity between the query vector and all key vectors *K*, which is used to weight the associated numerical vector *V*.

Each block begins with LN to normalize patch embeddings *z*. Then, *z*, the input of the *SA* (see Figure 2), is projected into Query, Key, and Value, where $Q=zW^{Q}$, $K=zW^{K}$ and $V=zW^{V}$ via $W^{q}\in R^{D\times D_{h}}$, $W^{k}\in R^{D\times D_{h}}$, and $W^{v}\in R^{D\times D_{h}}$. The corresponding attention weight can be written as

(2) $$A=softmax\left(\frac{{QK}^{T}}{\sqrt{d_{k}}}\right)$$

The output of *SA*, a product of the *A* and *V* matrices, can be written as:

(3) $$SA\left(z\right)=AV=softmax\left(\frac{{QK}^{T}}{\sqrt{d_{k}}}\right)V$$

*MHSA* computes attention scores, captures interactions between patches, and captures a larger range of correlation features by utilizing the *SA* mechanism multiple times, as known as multiple heads. If the number of heads is *k* and the self-attention of the *i*-th head is $SA_{i}\left(z\right)$, the following equation is obtained:

(4) $$\begin{array}{c} MHSA\left(z\right)=[SA_{1}\left(z\right);SA_{2}\left(z\right);\cdots ;SA_{k}\left(z\right)]W^{0}\\ MHSA\left(z\right)\in R^{N\times D} \end{array}$$

The output of *MHSA* is combined with the original embeddings *z* through a residual connection and normalized again. The embeddings are processed by an MLP, consisting of a feed-forward neural network (FFN) with ReLU activation, then followed by another residual addition and normalization. The output of the transformer encoder can be written as follows:

(5) $$\begin{array}{c} z_{l}^{\prime }=LayerNorm\left(z_{l-1}+MHSA\left(z\right)\right)\\ z_{l}=LayerNorm\left(z_{l}^{\prime }+FFN\left(z_{l}^{\prime }\right)\right) \end{array}$$

Data flow through the transformer encoder by sequentially going through each block. The output of one block becomes the input of the next block. After passing through the transformer encoder at the L layer, the final output is obtained:

(6) $$z_{L}=\left[z_{L}^{\left[cls\right]};z_{L}^{1};z_{L}^{1};\cdots ;z_{L}^{N}\right]$$

where $z_{L}^{\left[cls\right]}$ is a token used for classification, and *L* is the twelfth transformer encoder.

### 3.2. Schizophrenia Detection

After the transformer encoder module, the sequence of feature embeddings captures the semantic information of the input face images. These embeddings are aggregated and fed into an MLP for a classification task of detecting SZ. The MLP consists of four fully connected layers with ReLU activation. The result of the MLP can not tell people reliably whether the sample is schizophrenic, so it will be put into another softmax function, which outputs a probability score that indicates the likelihood more accurately.

Throughout the entire detection process, Binary Cross-Entropy (BCE) loss is applied to optimize the network. The BCE loss helps in minimizing the difference between the predicted probability score and the actual label, encouraging the model to produce accurate predictions.

The BCE loss function is defined as follows:

(7) $$L=-\frac{1}{N}\sum_{i=1}^{N}\left[y_{i}log\left({\hat{y}}_{i}\right)+\left(1-y_{i}\right)log\left(1-{\hat{y}}_{i}\right)\right]$$

where *N* is the number of training samples, $y_{i}$ is the ground truth label for the *i*-th sample (0 or 1), and ${\hat{y}}_{i}$ is the predicted probability for the *i*-th sample.

### 3.3. Semantic Feature Representation Visualization

ViT model is originally a transformer architecture, and uses self-attention mechanisms to capture relationships across the entire image. Therefore, the attention rollout technique is suitable to aggregate attention maps across the layers and heads of the ViT to identify significant regions in the input image.

For a given input image, the ViT model computes attention scores in each layer and head. Let $A^{\left(l\right)}$ denote the attention score matrix at layer *l*, where l=1, 2,⋯,L is the total number of layers. Each matrix $A^{\left(l\right)}$ is of $\left(n,n\right)$ dimension, where *n* is the number of tokens, including class tokens [*CLS*]s.

The attention rollout aggregates the attention score matrices across layers to form a unified attention map so that the contribution of each token at layer *l* will be traced back to the input tokens. The overall attention at the final layer *L*, denoted as $A_{rollout}$, is computed by multiplying the attention matrices from each layer:

(8) $$A_{rollout}=\prod_{l=1}^{L}{\tilde{A}}^{\left(l\right)}$$

where ${\tilde{A}}^{\left(l\right)}$ represents the normalized attention matrix at layer *l* as below, eliminating differences between layers.

(9) $${\tilde{A}}^{\left(l\right)}=\frac{A^{\left(l\right)}}{\sum_{i=1}^{n}A_{ij}^{\left(l\right)}}$$

Attention scores specific to the [*CLS*] token are instrumental in extracting the attention specific to the input image and therefore provide a comprehensive view of the regions influencing the model’s decision. Let $A_{rollout,\left[CLS\right]}$ denote the final attention map for the [*CLS*] token:

(10) $$A_{rollout,\left[CLS\right]}=A_{rollout,\left[CLS\right]}\cdot x$$

where *x* is the input image or sequence of tokens.

The attention map $A_{rollout,\left[CLS\right]}$ is designed to be visualized as a heatmap and overlaid on the original image to highlight the regions most influential for the model’s predictions, so it is processed by the next two steps:

The first is to resize the attention map into the same dimension of the original image as ${\hat{A}}_{rollout,\left[CLS\right]}$:

(11) $${\hat{A}}_{rollout,\left[CLS\right]}=resize(A_{rollout,\left[CLS\right]},(H,W))$$

To obtain better visualization results, the second step is to design the proportionality coefficient when overlaying the resized heatmap on the original face diagnosis image to visualize important regions explicitly:

(12) $$I_{overlay}=\cdot {\hat{A}}_{rollout,\left[CLS\right]}+(1-\alpha )I_{input}$$

where the α is set to 0.5.

In this way, the overlapping image can provide insights into which parts of the image contribute to SZ detection.

### 3.4. Analysis of the Importance of Clinical Facial Regions

First, the likelihood results of SZ detection derived from the ViT-based model are shown. Then, the eyes, mouth, nose, cheeks, and forehead areas of the face are segmented from the whole face by Mediapipe [35], and each is calculated to obtain the weight values among all the regions so as to help us understand the importance of each area of interest in detection. Finally, doctors will be provided with these two results. They can then analyze whether each sample suffers from SZ and tell the relationship between SZ and the representation of key semantic features in face diagnosis images.

Assisted with all this information, they can not only diagnose the condition of the current patient but also give more scientific and quantitative advice.

### 3.5. Participants

Schizophrenia group: patients diagnosed with schizophrenia were eligible for study inclusion.

Inclusion criteria included the following: (1) adults aged between 18 and 60 years. (2) Diagnosed with SZ according to the third edition of the Chinese Classification of Mental Disorders (CCMD-3) [36], verified by qualified psychiatrists. (3) Under stable antipsychotic treatment (e.g., olanzapine, clozapine, risperidone, or quetiapine) for at least three months. (4) Able to complete all study-related procedures, including facial image collection and other necessary evaluations. (5) Both the patients and their legal guardians provided informed consent after receiving a full explanation of the study’s purpose and procedures.

Exclusion criteria included the following: (1) use of antipsychotic medication for less than three months. (2) Presence of chronic central nervous system disorders (e.g., epilepsy, stroke) or severe physical illnesses that could confound the study results. (3) High risk of suicide or self-harm as assessed by a psychiatrist. (4) History of facial plastic surgery that could alter natural facial features. (5) Pregnant or breastfeeding women.

Healthy control group: healthy participants without psychiatric conditions were recruited as controls based on the following criteria.

Inclusion criteria included the following: (1) adults aged between 18 and 60 years. (2) No history of psychiatric illness or treatment for mental disorders. (3) No history of severe physical illnesses or major surgeries in the three months preceding enrollment. (4) Voluntarily agreed to participate in the study and provided written informed consent. (5) Demonstrated good compliance with study procedures and were able to cooperate during facial image collection.

Exclusion criteria included the following: (1) History of psychiatric medication use. (2) Pregnant or breastfeeding women. (3) Presence of makeup or facial modifications (e.g., scars or tattoos) that could interfere with accurate facial image analysis. (4) History of facial plastic surgery that could alter natural facial features.

All participants provided informed consent, and the study was approved by the institutional ethics committee.

### 3.6. Data Collection

**Figure 3:**
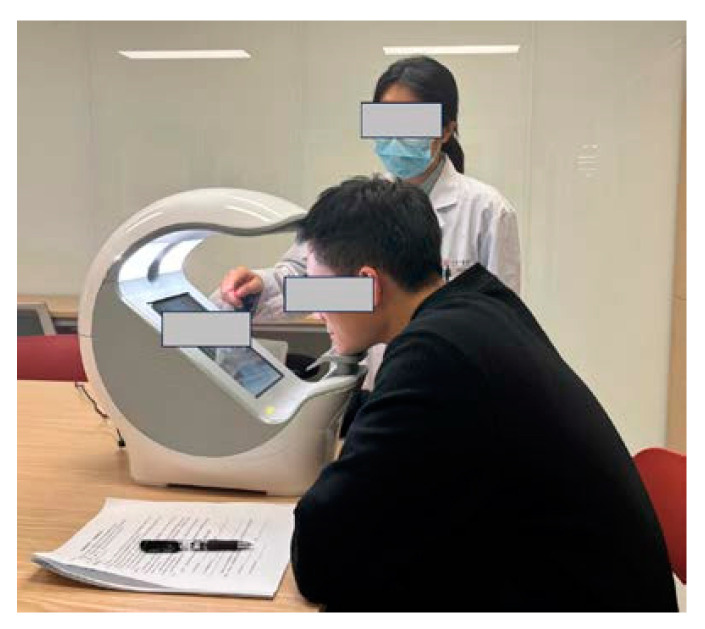
Data Collection Procedure.

A portable intelligent TCM diagnostic device independently developed by the Shanghai University of Traditional Chinese Medicine (model: TYJK201911D-B, patent numbers: ZL201921720435.8, ZL201930553056.3) [37,38] was used as the sampling instrument. As shown in Figure 3, participants were informed of the precautions to be taken prior to image acquisition, including avoiding makeup, removing glasses, and ensuring that hair did not cover the forehead. After reading and signing a written informed consent form, they were seated in a sitting position in front of the device, placing their chin on the chin rest and their forehead gently against the upper support. During acquisition, participants were asked to remain still, face the screen, and ensure that their entire face was located within the designated frame. Frontal facial images were then captured and stored securely for further analysis. General demographic information, including sex, age, height, weight, and other relevant details, was collected for each participant. These data were recorded and saved in an Excel document for subsequent statistical analysis. To ensure data consistency, the same image acquisition procedure was followed for both the schizophrenia group and the healthy control group.

### 3.7. Statistical Analysis

Continuous variables, such as age, height, and weight, were analyzed using independent t-tests, and results are presented as means ± standard deviations. Categorical variables, such as sex, were analyzed using the Chi-square test. All comparisons were two-sided, with statistical significance defined as *p* < 0.05. Analyses were calculated using SPSS version 26.0.

## 4. Results

### 4.1. Experimental Setup

The SZ Dataset totally includes 921 face diagnosis images separated from 505 SZ patients and 416 healthy participants. Face diagnosis images were captured by professional equipment in an open environment. The original image size is 480 × 640 pixels. It is guaranteed that the image acquisition study follows the requirement of the Ethics Committee of Putuo District Center Hospital (No. PTEC-A-2024-50(S)-1).

Image processing includes face segmentation and resize operation. Mediapipe, a face segment method [35], keeps the shape of the original images and eliminates irrelevant objects (such as hair and neck) and environmental background from them, increasing the accuracy of SZ detection and reducing the amount of calculation. After segmentation, the original images of 480 × 640 are resized to *H* × *W* = 224 × 224 to meet the input requirements of ViT inputs.

Metrics. Four widely used metrics are adopted for model evaluation.

(13) $$Accuracy=\frac{TP+TN}{TP+TN+FP+FN}$$

(14) $$Precision=\frac{TP}{TP+FP}$$

(15) $$Recall=\frac{TP}{TP+FN}$$

(16) $$F1-Score=\frac{2\times Precision\times Recall}{Precision+Recall}$$

Hardware condition. The experiments were implemented by Python 3.9.18 and PyTorch 2.3.0 in the CUDA 11.8 universal computing framework, running on an Ubuntu 20.04.1 operating system with one NVIDIA 3090 GPU with 24 GB memory.

Model parameter settings. The data were trained in 100 epochs with a learning rate of 0.001 and a batch size of 128. The optimizer is Adam [39] with betas of (0.9, 0.999). Additionally, five-fold cross-validation was employed to make full use of the data and reflect the stability and reliability of the models. In each fold, 80% of the dataset was used for training, while the remaining 20% was used for validation and testing.

### 4.2. Study Population

We collected clinical information for all participants, including age, sex, height, and weight. The baseline characteristics of the patient group (*n* = 505) and the healthy control group (*n* = 416) are summarized in Table 2. No statistically significant differences were observed between the two groups in sex, age, height, and weight, so the two groups are comparable and suitable for subsequent analyses.

All participants in this study were of Asian ethnicity. Among the patient group, all individuals were receiving antipsychotic medication at the time of image collection. In contrast, healthy participants had no history of psychological disorders and had never taken psychiatric medications.

### 4.3. Detection Result Evaluation

The performance of the ViT-based model for SZ detection will be compared with these representative machine learning methods in the unified environment described above: SVM [40], ResNet34 [41], ResNet50 [41], VGG19 [42], and DenseNet121 [43].

SVM is a classical machine learning algorithm used for classification tasks. ResNet is a deep neural network architecture that introduces residual connections to help mitigate the vanishing gradient problem in deep neural networks. VGG is a family of convolutional neural networks known for their deep architectures with small receptive fields (3 × 3 convolutional layers). DenseNet connects each layer to every other layer in a feed-forward fashion, resulting in a network with fewer parameters and more efficient gradient flow. These models are commonly used in image classification tasks and serve as benchmarks for evaluating the effectiveness of our proposed ViT-based approach.

In addition to the above models, the large multimodel modal BLIP-2 [44] is also introduced in baseline comparison. BLIP-2 is designed to bridge language and vision tasks and to realize multimodal understanding and generation. Given that it is supposed to be applied in our unified experimental environment to ensure consistency, its downstream function of feature extraction is chosen to insert into our model and replace the semantic feature representation part in it.

As shown in Table 3, the results demonstrated the accuracy superiority of our model over traditional machine learning methods and neural network architectures for SZ detection. The accuracy scores of SVM and VGG19 were 77.72% and 78.05%, respectively. Our model achieved an accuracy of 97.83%, outperforming all baseline models, including BLIP-2, which recorded the highest accuracy among them at 94.14%. Not only that, our modal performed better than other baseline models, which validates its effectiveness as a learning feature representation for this task. The precision, recall, and F1-Score of our model were over 97%, indicating its robust ability to accurately detect SZ with minimal errors.

**Figure 4:**
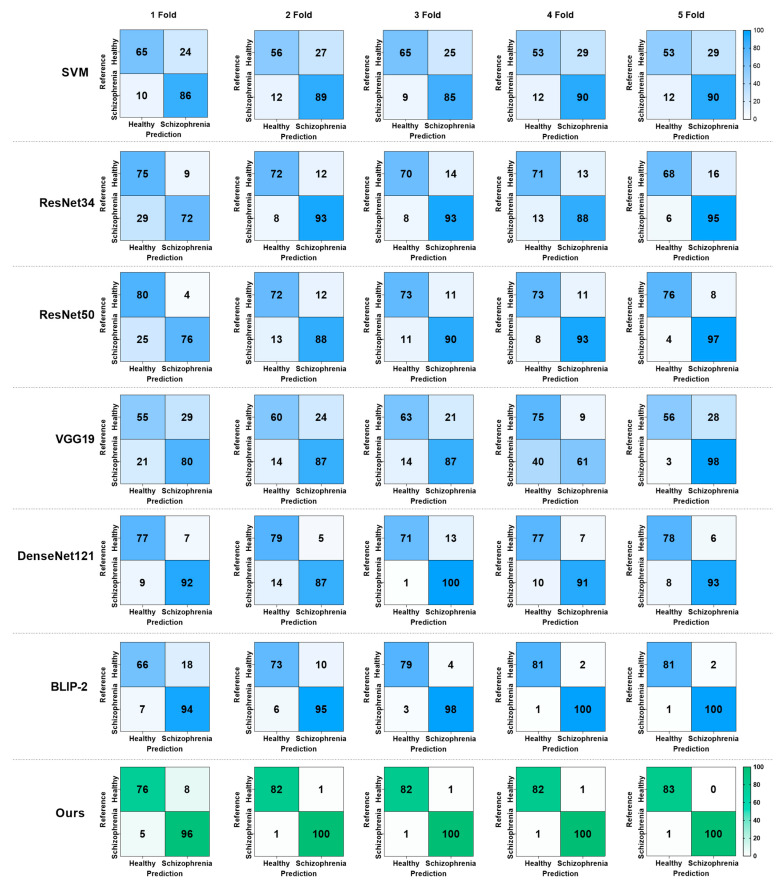
The five cross-validation of confusion matrix result of models. From top to bottom, they are SVM, ResNet34, ResNet50, VGG19, DenseNet121, BLIP-2, and our method. From left to right, a confusion matrix from one fold to five folds.

Further, the confusion matrix showed the five-fold cross-validation results of different models, as shown in Figure 4. It is demonstrated that SVM performed poorly because approximately one-third of the healthy individuals were misclassified as SZ patients. The performance of VGG19 was similar to that of SVM, and many healthy individuals were incorrectly detected as SZ patients, affecting the overall accuracy. The results of ResNet34 and ResNet50 achieved high accuracy and demonstrated good detection ability, but some healthy individuals were still misclassified as SZ patients, possibly due to the model’s insufficient precision in identifying facial feature representation in healthy individuals. The confusion matrix of BLIP-2 showed a significant reduction in the number of misclassifications, indicating greater reliability in identifying healthy individuals and SZ patients. Our proposed model achieved the best performance in all tests. In the five-fold cross-validation, particularly in folds 2 to 4, only two face diagnosis images were misclassified. This demonstrates the exceptional capability of our model in distinguishing between healthy individuals and SZ patients.

### 4.4. Visualization Comparison

**Figure 5:**
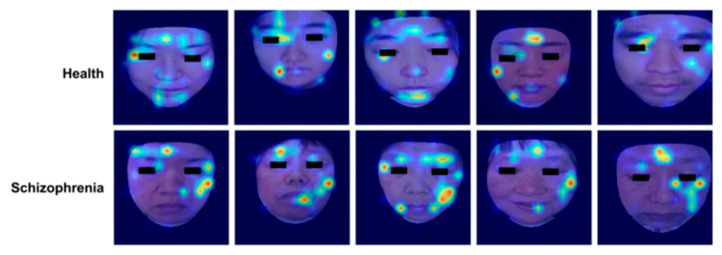
Some examples of facial visualizations of representations of important facial features. The top row is healthy participants, and the bottom row is SZ patients. Red and blue indicate high and low attention, respectively.

Figure 5 shows several cases where the attention rollout method was employed to visualize the face diagnosis images of healthy people and SZ patients. The generated heatmaps indicated the focus of the model, with red areas signifying high attention and bluer areas indicating low attention. It is evident that the eye and mouth regions are most focused on. This confirms that these areas are crucial for detecting SZ. Interestingly, the forehead and cheek areas also show significant contributions.

We already know that attention maps generated by the model highlight the key facial regions contributing to its classifications. In the case of correct classifications, the model focused primarily on the eyes and mouth regions for both healthy individuals and schizophrenia patients, which aligns with clinical observations that these areas carry important diagnostic cues.

**Figure 6:**
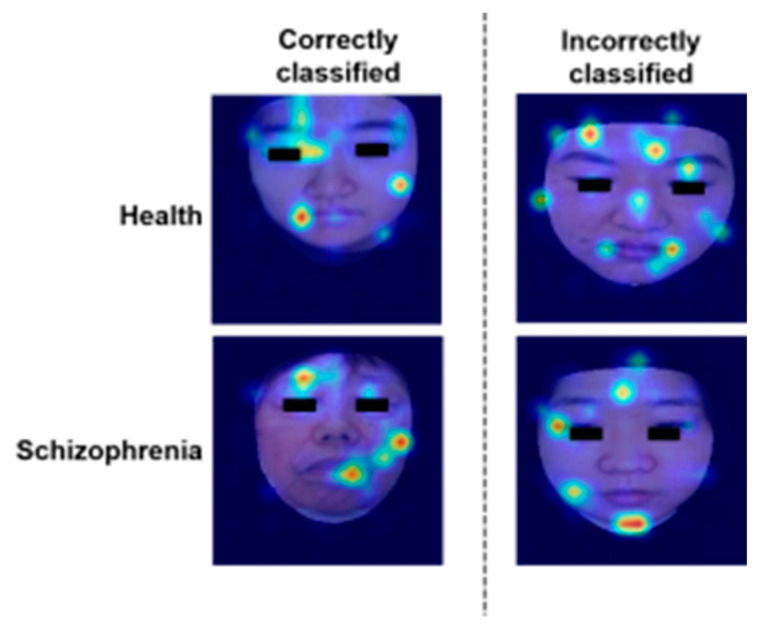
Examples of correctly and incorrectly classified cases visualized using the attention rollout method. The top row represents healthy participants, and the bottom row represents SZ patients. Red and blue indicate high and low attention, respectively.

However, for incorrect classifications, the model incorrectly prioritized features such as the cheeks or exhibited scattered attention across the face. Figure 6 highlights specific examples of correct and incorrect classifications for both healthy participants and SZ patients. These visualizations illustrate how the model identifies key facial regions in correct classifications but occasionally shifts its focus to less relevant areas in misclassified cases. These examples illustrate both the strengths of the model in capturing meaningful patterns and its limitations in handling certain edge cases.

### 4.5. Clinical Facial Features Analysis

**Figure 7:**
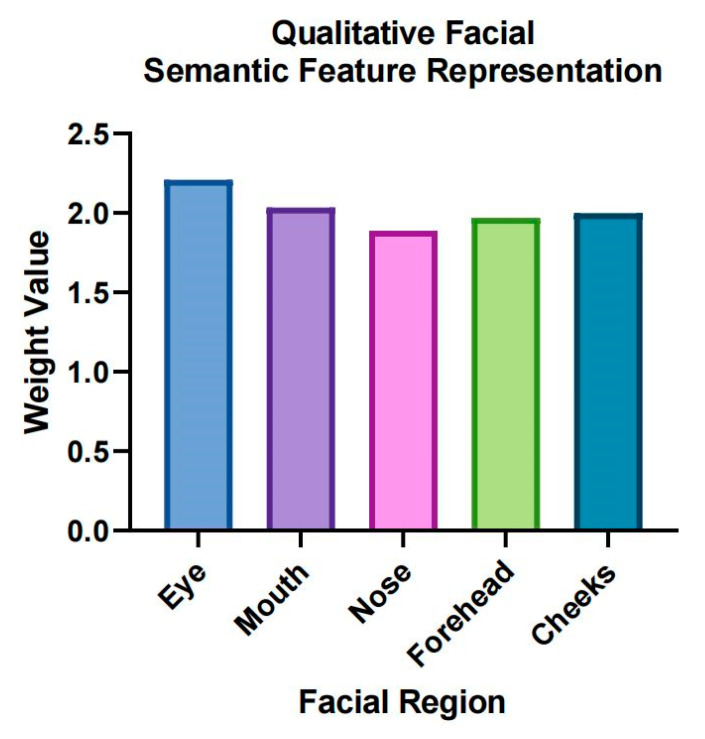
Weight values of facial semantic feature representation in five different regions: eyes, mouth, cheeks, nose, and forehead.

To provide a deeper insight into the SZ detection process, the semantic feature representations of key facial regions were analyzed. The weights assigned to each facial region are shown in Figure 7.

These weights reflect the relative importance of each facial region in contributing to the model detection. It was found that the order of importance of facial features in SZ detection was: eyes, mouth, forehead, cheeks, and nose. The eye region received the highest weight, indicating that feature representations learning from this area were most influential in distinguishing between SZ patients and healthy individuals. This finding aligns with clinical observations, where eye movement and expression are often noted as significant indicators of SZ.

## 5. Discussion

As we can see from the figures above, by combining detection results and feature importance analysis, the model not only enhances the transparency and interpretability in the SZ detection field but also provides a standard, objective, and supportive approach for clinicians to get valuable supplementary information.

For one thing, we acquire the areas of the face on which the detection model is focused (Figure 5). With an attention mechanism, the model can give each facial feature a weight in SZ detection. By highlighting regions with high weights, we help clinicians understand which facial regions play a key role in the detection model and make a final diagnosis with clinical experience.

Figure 6 further illustrates examples of both correctly and incorrectly classified cases for healthy participants and SZ patients. In correctly classified cases, the model demonstrates a consistent focus on the eye and mouth regions, aligning with known clinical features indicative of SZ. For instance, a correctly classified SZ patient (bottom left image) shows heightened attention around the eyes and mouth, which are critical regions for diagnosing mental conditions.

However, in incorrectly classified cases, the model sometimes shifts its attention to less relevant areas, such as the cheeks or forehead, or fails to focus adequately on critical regions. For example, a healthy participant (Figure 6, top right image) was misclassified as schizophrenic due to sad facial expressions, which introduced noise into the attention mechanism. Similarly, a misclassified SZ patient (Figure 6, bottom right image) exhibited atypical features, likely influenced by medication side effects.

These examples highlight both the strengths and limitations of the model. Attention rollout enhances interpretability by visualizing key regions, but the model’s reliance on specific features can lead to errors in cases with external confounding factors. Addressing these limitations through additional data preprocessing or incorporating robustness against noise will further improve the model’s performance.

For another, we quantify the contribution of areas such as the eyes, mouth, forehead, cheeks, and nose during the detection process (Figure 7). The results of the importance analysis of facial features showed that the eye area was the most important in the detection, followed by the mouth and forehead, while the cheeks and nose contributed less.

In this way, clinicians can obtain a more comprehensive understanding of the patient’s psychological condition and make more accurate diagnostic decisions. This model is expected to be widely used in clinical practice to provide stronger support for the SZ diagnosis.

Moreover, unlike traditional feature extraction, the model leverages semantic feature representation learning from facial images based on a pre-trained ViT model and gains a high level of accuracy in detecting SZ. It suggests that the ViT-based model effectively captures relevant features for distinguishing between schizophrenic and non-schizophrenic individuals. Furthermore, as shown in the last chapter, the proportion of false positives and false positives can be decreased to a great extent compared with other models, which reduces unnecessary anxieties and misdiagnoses for patients, respectively.

From the perspective of its application, TCM owns a theory called “preventive treatment of disease”, which means taking appropriate measures to maintain health and prevent the occurrence and progression of diseases. So it is of great significance to seek effective and interpretable methods for SZ detection, but previous SZ detection implemented by advanced modern medical equipment is too complicated when used and usually provides only a simple test result, thus causing inconvenience in patients’ examination and not able to offer perspectives of TCM.

The interpretability provided by attention maps also reduces the training requirements for clinicians. With minimal training, healthcare professionals can understand the visual outputs, such as the model’s focus on key facial regions, and integrate these insights into their diagnostic workflow. This interpretability enhances the model’s utility as a supportive tool in clinical practice, complementing existing diagnostic approaches.

Face diagnosis images can be captured using standard cameras or mobile devices, making the method accessible and practical even in low-resource settings. Furthermore, the reduced dependence on expensive equipment such as MRI or EEG systems lowers the overall cost of implementation, allowing this model to be integrated into a wide range of clinical environments.

This study proposes a novel model that firstly uses face diagnosis images as direct objects of feature representations and provides SZ detection results accurately, along with a detailed visualization of facial features, which can be interpreted and analyzed by prior knowledge of TCM.

In the future, we need to optimize the performance of the model, increase more facial features for analysis, and enhance the generalization ability and accuracy of the model. Additionally, the application of the model in other mental diseases can be explored to further verify its effectiveness and scalability.

## 6. Conclusions

This study demonstrated that the proposed ViT-based model provides high accuracy and interpretability in SZ detection, outperforming benchmark models in evaluation metrics. The model can quantify feature importance in facial regions and offer new insights for clinical application. The integration of facial feature analysis into SZ detection not only enhances detection accuracy but also bridges the gap between clinical appliance and data-driven insights, offering a robust tool for early intervention in mental health care. Future research should optimize and improve the generalization ability of the model and explore its potential in different patient populations to fully realize its clinical utility.

## Figures and Tables

**Table 1:** Comparison of SZ Detection Methods.

Method	Invasiveness	Clinical Interpretability	Patient Compliance	Key Advancement	References
EEG	High	Low	Medium	Requires cooperation, difficult to interpret	[8,9]
MRI	High	Low	Low	Expensive, time-consuming, but highly accurate	[6,7]
Face Diagnosis Image	Low	High	High	Novel non-invasive AI method leveraging ViT for SZ detection	Current study

**Table 2:** Baseline characteristics.

Group	Count	Sex [Count (Percentage)]		Age (Year)	Height (cm)	Weight (kg)
		Male	Female			
Patient	505	266 (52.67%)	239 (47.33%)	46.84 ± 7.01	165.56 ± 8.25	70.77 ± 13.50
Healthy	416	227 (54.57%)	189 (45.43%)	39.68 ± 7.40	166.45 ± 7.97	62.90 ± 12.60
*p*-Value		0.57		0.62	0.31	0.37

**Table 3:** Evaluation metrics results for seven models.

Model	Accuracy	Precision	Recall	F1-Score
SVM	77.72%	78.58%	76.43%	76.35%
ResNet34	86.16%	86.62%	86.16%	86.12%
ResNet50	88.43%	88.88%	88.43%	88.42%
VGG19	78.05%	79.28%	78.05%	77.79%
DenseNet121	91.35%	91.60%	91.35%	91.35%
BLIP-2	94.14%	94.11%	94.23%	94.14%
Ours	97.83%	97.84%	97.83%	97.83%

## Data Availability

The data presented in this study are available upon request from the corresponding author due to privacy.
